# BioAssay Ontology (BAO): a semantic description of bioassays and high-throughput screening results

**DOI:** 10.1186/1471-2105-12-257

**Published:** 2011-06-24

**Authors:** Ubbo Visser, Saminda Abeyruwan, Uma Vempati, Robin P Smith, Vance Lemmon, Stephan C Schürer

**Affiliations:** 1 Department of Computer Science, University of Miami, Coral Gables, FL, USA; 2 Center for Computational Science, University of Miami, Miami, FL, USA; 3 The Miami Project to Cure Paralysis, Department of Neurological Surgery, University of Miami, Miami, FL, USA; 4 Department of Molecular and Cellular Pharmacology, Miller School of Medicine, University of Miami, Miami, FL, USA

## Abstract

**Background:**

High-throughput screening (HTS) is one of the main strategies to identify novel entry points for the development of small molecule chemical probes and drugs and is now commonly accessible to public sector research. Large amounts of data generated in HTS campaigns are submitted to public repositories such as PubChem, which is growing at an exponential rate. The diversity and quantity of available HTS assays and screening results pose enormous challenges to organizing, standardizing, integrating, and analyzing the datasets and thus to maximize the scientific and ultimately the public health impact of the huge investments made to implement public sector HTS capabilities. Novel approaches to organize, standardize and access HTS data are required to address these challenges.

**Results:**

We developed the first ontology to describe HTS experiments and screening results using expressive description logic. The BioAssay Ontology (BAO) serves as a foundation for the standardization of HTS assays and data and as a semantic knowledge model. In this paper we show important examples of formalizing HTS domain knowledge and we point out the advantages of this approach. The ontology is available online at the NCBO bioportal http://bioportal.bioontology.org/ontologies/44531.

**Conclusions:**

After a large manual curation effort, we loaded BAO-mapped data triples into a RDF database store and used a reasoner in several case studies to demonstrate the benefits of formalized domain knowledge representation in BAO. The examples illustrate semantic querying capabilities where BAO enables the retrieval of inferred search results that are relevant to a given query, but are not explicitly defined. BAO thus opens new functionality for annotating, querying, and analyzing HTS datasets and the potential for discovering new knowledge by means of inference.

## Background

High-throughput screening (HTS) has evolved into an industrialized process and HTS of small molecules is one of the most important strategies to identify novel entry points for drug discovery projects [1]. Until about half a decade ago, HTS and ultra-high throughput screening (uHTS) have been primarily in the realm of the pharmaceutical industry where huge amounts of data have been generated using these technologies. In 2003, NIH started to make HTS and uHTS capabilities accessible to public sector research via the Molecular Libraries Initiative [2] to advance translational research [3] and specifically the Molecular Libraries Program (MLP) [4]. MLP projects leverage innovative assay technologies to develop compounds effective at modulating biological processes or disease states via novel targets. The program has established publicly funded screening centers along with a common screening library (the MLSMR, Molecular Libraries Small Molecule Repository) and data repository, PubChem [5]. Following a pilot phase, the Molecular Libraries Probe Production Centers Network (MLPCN), which consists of four comprehensive and three specialized centers, has been running numerous screening campaigns and has produced a wide range of chemical probes [6]. Since 2004, the MLPCN centers have deposited over two thousand HTS assays testing the effects of several hundred thousand compounds. More recently a European effort, EU Openscreen [7], to establish small molecule screening capabilities is being developed. Besides PubChem there are other data repositories including ChEMBL [8], which includes data curated from the medicinal chemistry literature, and the Psychoactive Drug Screening Program (PDSP) [9] with mostly receptor and ion channel binding assay results. The MLP is currently the largest public screening effort. The pace with which novel biological assay and HTS results are being submitted suggests that we have only begun to explore the scope of possible assay formats and technologies to interrogate complex biological systems.

Similar to the HTS datasets produced in the pharmaceutical industry, the public sector screening data represent an invaluable resource, which has received wide-spread attention (including from the pharmaceutical companies). However, their diversity and quantity also present enormous challenges to organizing, standardizing, and integrating the data with the goal to maximize their scientific and ultimately their public health impact as the screening results are carried forward into drug development programs. Despite calls for HTS standards [10], there have been no public initiatives defining minimum specifications, data exchange formats, or a controlled terminology. This situation lies in contrast to other fields such as microarray experimentation, where minimum information specifications (Minimum information about a Microarray Experiment or MIAME 2.0), multiple data models (MicroArray Gene Expression Object Model or MAGE-OM) and the MGED (Microarray and Gene Expression Data) ontology [11] have been developed and incorporated into Web Services such as the Gene Expression Omnibus [12] to facilitate data exchange. PubChem [13] was set up with flexibility in mind and is able to collect almost any type of assay results. Screened compounds and substances are represented seamlessly by chemical structure files and pertinent assay data are interlinked to other NCBI resources. However, PubChem has limitations that burden data retrieval and meta-analysis. Foremost is an unstructured/semi-structured data representation format that is largely determined by the submitter. Information regarding assay formats (e.g. cell-based vs. biochemical), readout technologies, reagents employed, and details of the biological system interrogated are represented as free text. This makes it impossible to query PubChem by simple, yet relevant concepts, such as "*luciferase reporter gene *assays" or "*GPCR agonist *assays".

To describe compound activities, PubChem uses two terms, "Outcome" and "Score", that have different connotations depending on the submitter. This discrepancy effectively renders quantitative comparisons between assays impossible. Additional terms describing assay results (referred to as assay endpoints in this paper), such as the half maximal inhibitory concentration (IC50), have different nomenclatures. For example, "JAK2V617F Inhibition (IC50) " (AID 2165), "mutant luminescence Mean IC50" (AID 792), "Best-Fit Value IC50 (uM) " (AID 1916), "IC50_Mean" (AID 2784), "Mean IC50" (AID 1695) are all equivalent endpoints for the purpose of analysis, but are distinct in the repository. This system has led to the accumulation of over 17,000 unique endpoints that cannot be compared without large-scale annotation efforts. In addition to inconsistent naming, there is no semantic description of screening endpoints. In this paper, we show how the definition of endpoints (such as "IC50") in an ontology with formal semantics facilitates the retrieval of data that are relevant to a search query, but not explicitly defined by the query terms (inferred results).

Ontologies have traditionally been used in biology to organize information within a domain and, to a lesser extent, to annotate experimental data. A successful and highly-used biomedical ontology is the Gene Ontology (GO) [14], which consists of a taxonomy of terms describing gene product localization and function. Several hundred ontologies are hosted by the Open Biological and Biomedical Ontologies (OBO) Foundry (102, counted on 4/15/2011) [15] as well as the National Center for Biomedical Ontologies (NCBO, 263 ontologies counted on 4/15/2011) [16], centered on domains ranging from African traditional medicine to Zebra fish anatomy. A closer look reveals that the majority of these ontologies are actually taxonomies or "enriched taxonomies" (with comments for understanding). It has been suggested that the general utility of many of these ontologies is likely overestimated, because terms lack clear semantics and multiple conventions are used to describe overlapping information [17]. In addition, many of the biomedical ontologies so far have not made use of available description logic (DL) features of the Web Ontology Language (OWL), the official ontology language recommended by the World Wide Web Consortium (W3C).

In this paper we describe a novel approach to standardize, organize and semantically define biological assays and screening results such as those in PubChem, and which addresses many of the challenges raised above. We briefly discuss the main components required to describe important details of bioassay experiments and screening results. We illustrate the architecture of the BioAssay Ontology (BAO) and show examples of how some of the concepts are implemented in BAO to serve as a standard and as a knowledge model.

BAO is organized by several main concepts, which describe important characteristics of assays and by which assays can be meaningfully categorized. One of the goals of BAO is to enable the classification of assays by relevant categories so that related assays can quickly be identified, for the purpose of data analysis or assay development [18]. These main categories relate to questions like: i) What type of perturbing agent (perturbagen) was screened? ii) What was the main biological/chemical category (format) of the assay? iii) How was the perturbation converted into a detectable signal? iv) What was the physicochemical method of signal detection? v) What was the biological context (meta target) of the assay? vi) How were the results reported/quantified?

A full description of BAO is beyond the scope of this paper and will be reported elsewhere. For details we refer to the BAO website [19]. A novel feature of BAO is that it supports inferences within the functionality of OWL 2.0, raising the possibility of automated knowledge acquisition from existing datasets. We present a number of semantic query scenarios that are enabled by BAO. In addition to identifying assays and data by concepts in the ontology, we show how our approach can retrieve inferred results that are highly relevant to a query, but would not match the search term explicitly and therefore could not be easily identified by a classical (relational) search. These type of queries are made possible by the standardization that is provided via BAO and the reasoning/inference capabilities of the system.

## Results and Discussion

### Main concepts of the BioAssay Ontology and curation of PubChem assays

BAO describes biological screening assays, in which the perturbation of a biological system or a component thereof (relative to a reference state) by a perturbagen is detected and in many cases quantified. An example for a simple assay is the inhibition of an enzyme by a small molecule, which would be detectable by quantifying the product of the enzymatic reaction. For example inhibition of a kinase could be detected via an antibody specific to the phosphorylated substrate (a kinase catalyzes the phosphorylation of a substrate by ATP). In one assay design, the antibody is linked with a fluorescence resonance energy transfer (FRET) donor and the (kinase) substrate with a FRET acceptor. A fluorescence signal of the FRET acceptor is only generated if donor and acceptor are in proximity, i.e. if the substrate is phosphorylated. If the kinase is inhibited by a small molecule perturbagen, the signal decreases. An implementation as homogeneous time resolved FRET (HTRF) assay is applicable to high throughput screening. Countless sophisticated biological screening assays to interrogate simple to complex biological systems have been developed.

With BAO we aim to develop an open standard for the description of HTS and microscopy-based high-content screening (HCS) assays and data for the purpose of classification and analysis. To describe biological screening experiments such as those deposited in PubChem, we first identified the main categories that need to be captured in order to meaningfully compare data from different biological screening experiment. These components are perturbagen, format, design, detection technology, meta target, endpoint, which are described here:

#### Perturbagen

Assay "perturbagen" refers to the agent that directly interacts or indirectly affects the meta target of a bioassay. PubChem assays predominantly have small molecules as perturbagens; however the concept perturbagen in BAO includes various other perturbing agents, including, nucleic acid (e.g. siRNA, cDNA), lipid, or proteins. Perturbagen specifications include perturbagen source and details on its delivery.

#### Assay Format

The assay "format" is a higher-level assay category that relates to the biological and chemical features that are common to each test condition in the assay. Assay format includes several broad categories. "Biochemical format" describes assays that are performed with a purified protein, such as the example above. "Cell-based format" relates to assays that are performed with living cells. "Organism-based format" refers to assays with a living organism. Other common formats include "cell-free format", "tissue-based format", and "physicochemical format". Additional format specifications are captured that describe, for example, whether the assay is homogeneous or heterogeneous in nature.

#### Assay Design and Detection Technology

The assay "design" describes the methodology to report the action of the perturbagen on the target; i.e. how the perturbation is converted into a detectable signal. In BAO, assay design is broadly classified into one of eight categories: "binding reporter", "enzyme reporter", "inducible reporter", "morphology reporter", "viability reporter", "redistribution reporter", "conformation reporter", and "membrane potential reporter". We further annotated the readout "detection technology" used in the assays. These annotations fall into one of several categories, including "spectrophotometry", "fluorescence", "luminescence", "label free technology", "scintillation counting", and "microscopy". Further specifications of assay design and detection technology can include the assay kit or detected wavelength.

#### Assay Meta Target

Assay "meta target" is a description of the component(s) of the biological system that interact with the perturbagen. Meta target can be directly described as a molecular entity (e.g. a purified protein or a protein complex), or indirectly by a biological process or event (e.g. phosphorylation), or a signaling pathway. An important aspect of our meta target annotations is that they are embedded with semantic information (e.g. *"is target of" *only "measure group"; disjointness with classes such as "perturbagen" or "endpoint"). Meta target may be further linked to additional terms and external content, such as a pathway database. One of the goals of describing meta targets is to infer possible molecular targets or perturbagen mechanisms of action based on the analysis of results of many related assays. Meta target specifications include protein modifications, cell lines, or details about the mechanism of ligand-protein interaction.

#### Assay Endpoint

An assay "endpoint" describes a quantitative or qualitative outcome of the bioassay. The main classes that we identified are "perturbagen concentration"- and "response"-type endpoints. Simple examples include IC50, EC50, CC50 and percent inhibition, percent activation, percent viability, respectively. We conducted two stages of endpoint formalization, the first of which was to standardize the endpoint names in PubChem by manual curation. This reduces the number of different representations of each endpoint concept. In the examples illustrated below we have reduced 85 unique PubChem endpoint representations to 18 standardized endpoints. However, it is not possible (by manual curation) to uniquely describe each endpoint by exactly one representation, because the endpoint concept depends on other assay concepts and can even vary among different perturbagens of the same assay. In BAO, we therefore defined the endpoint concepts semantically using description logic to specify relationships among the endpoint types and other BAO concepts (see below, Ontology-facilitated query examples, example 3). This enables us to retrieve inferred results, which could otherwise not be obtained or would require complex Boolean endpoint queries. An excerpt of BAO around the class assay endpoint is shown below (Ontology outline, development and implementation).

For the purpose of demonstrating the semantic querying capabilities facilitated by BAO (which are described below) we curated over 300 bioassays from PubChem and standardized the endpoints using BAO.

### Ontology outline, development and implementation

BAO was designed to describe biological screening experiments and their outcomes by the six main components outlined above, in addition to general assay attributes that don't fall into any of these categories. Each BAO component includes multiple levels of sub-classes and specification classes which are linked via object property relationships to form a knowledge representation. A full description of this schema will be discussed elsewhere; the current version of the ontology (v1.2b868), is available on our website and at the NCBO bioportal. Our development approach follows established ontology engineering methodologies using a combination of top-down, domain expert-driven and bottom-up, data-driven approaches [20]. The current version of BAO consists of 730 OWL 2.0 [21] classes, 72 object properties (relations), 7 data properties, and 25 individuals (not including any annotated assays). Several external ontologies contain partial information of some of the components of biological assays described by BAO. To leverage these efforts, we have imported into BAO relevant sections from Gene Ontology (GO) [14], Cell Line Ontology (CLO) [22], Unit Ontology (UO) [23] and others. GO biological process terms and CLO cell line names and additional parameters are used in BAO meta target and meta target specifications. Organism names associated with targets were imported from NCBI taxonomy. Protein target names and IDs were referenced from UniProt. From UO we imported concentration unit and time unit terms. We are currently working on mapping BAO to other OBO ontologies. For example, OBI includes relevant information to describe biological assays [24]. We have mapped some of the BAO relationships to the OBO Relationship Ontology (RO) [25] and we aim to make more use of RO relationships in the future. Additionally, we may be able to use RO to map BAO concepts to other ontologies, in particular OBI. BAO is "rich" with a DL expressivity of ALCHOIQ(D). This means that the ontology has the basic S (ALC) expressivity [26] with role hierarchies (H), nominals (O), inverse properties (I), qualified cardinality restrictions (Q), and the use of datatype properties, data values or data types (D). It should be noted that three major bioinformatic terminology bases: SNOMED [27], Galen [28], and GO [14] have the expressivity of EL, with additional role properties. In EL, only intersections between concepts and full existential quantification are possible. In comparison, BAO is a significant improvement in expressivity.

**Figure 1:**
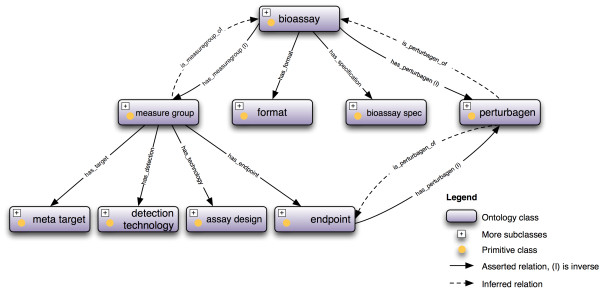
**BAO excerpt showing the root-level classes and some of their relationships**.

**Figure 2:**
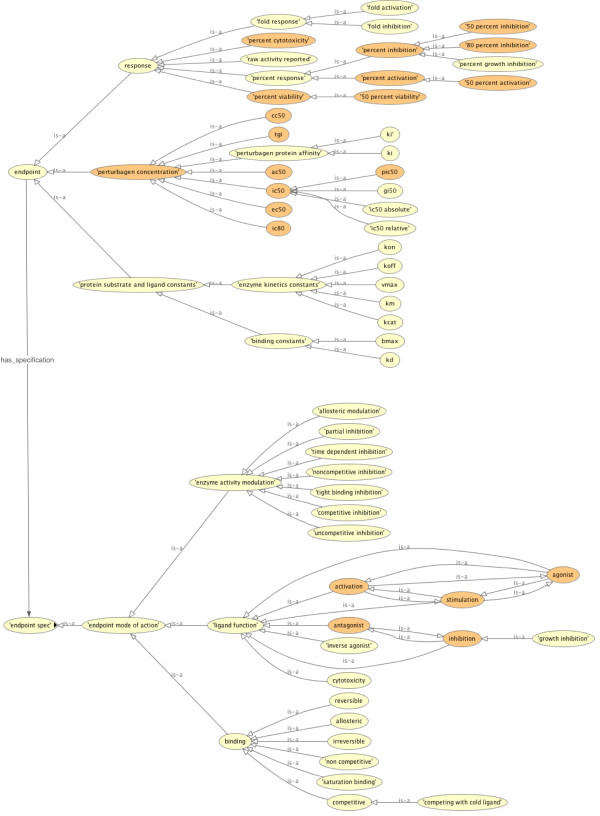
**A view on some of BAO's concepts, defined as either primitive (light gray/yellow) or defined classes (dark gray/orange)**.

Figure 1 illustrates the high-level outline of BAO. It shows the root-level classes, which are described above and general bioassay specifications, and some of their relationships. Some concepts (format, perturbagen and bioassay specifications) are linked directly to bioassay while others (endpoint, meta target, design, detection technology) are linked via a measure group to accommodate multiplexed and multi-parametric assays. It is also important to note that the assay components are not modeled as sub-classes of bioassay, because they do not have a formal "*is a*" relationship to bioassay. The bioassay component specification classes are not shown. Figure 2 shows an excerpt of the BAO classes (and their subsumption hierarchies) that are related to the concept "endpoint". For example Figure 2 illustrates the different type of endpoints, such as concentration- and response-type and also the relationships to the specification class, which includes (among others) "endpoint mode of action" with various sub-classes. These concepts are relevant for the semantic querying and reasoning capabilities described in the examples below.

The complete specification in OWL 2.0 can be visually explored and downloaded from our web page http://www.bioassayontology.org/visualize/. To illustrate how each of these classes is embedded with semantic information, the following example depicts a detailed specification for the class "IC50", defined as the concentration of the perturbagen that results in 50% inhibition.

### Equivalent classes

ic50 ≡ (∃"has has mode of action".inhibition) ⊓

(∀"has mode of action".inhibition) ⊓

("has percent response". "50 percent inhibition individual")

### Superclasses

ic50 ⊑ (∀"has curvefit spec". "curvefit spec")

ic50 ≡ "perturbagen concentration"

### Inherited anonymous classes

ic50 ⊑ (∃"has perturbagen concentration unit". "concentration unit") ⊓

(∀"has perturbagen concentration unit"."concentration unit") ⊓

(= 1"has perturbagen concentration value".xsd: float) ⊓

(∀"has specification"."endpoint spec") ⊓

(∃"has perturbagen".perturbagen) ⊓

(= 1"has perturbagen".T)

### Symbols

≡: equivalentClass, ⊑: subClassOf, ∀: allValuesFrom, ∃: someValuesFrom, = N: exactly N, T: Thing.

It is important to note that in OWL 2.0, there are only definitions for equivalent classes (necessary & sufficient conditions), and superclasses (necessary conditions). Necessary and sufficient conditions are used to classify individuals; for example we might be able to infer that an individual endpoint must be an IC50 because the mode of action is inhibition (among other criteria). With only necessary conditions, the definition is logically different, saying that if an individual is a member of the class IC50, it is necessarily a sub-class of "perturbagen concentration". The equivalent class IC50 specifies *"has mode of action" *only "inhibition". "Only" here denotes universal quantification, describing all the individuals whose *"has mode of action" *relationships refer to members of the class inhibition; or conversely, the individuals that do not have "has mode of action" relationships to individuals that are not members of the class "inhibition". There are also existential restrictions that can be seen as "among other things", and are used to close a given property, which is necessary for the reasoning process. The keyword "some" denotes existential restrictions. An example in our ontology is *"has mode of action" *some "inhibition". This specifies the existence of at least one relationship along a given property to an individual, which is a member of the class IC50.

Certain specifications are inherited from classes that are higher up in the class hierarchy. An example of this is the inherited anonymous class definition of individuals having the object property *"has perturbagen concentration value"*. There is also the relationship *"has perturbagen"*, describing that every individual of the IC50 class must have at least one perturbagen.

### Ontology implementation and application

**Figure 3:**
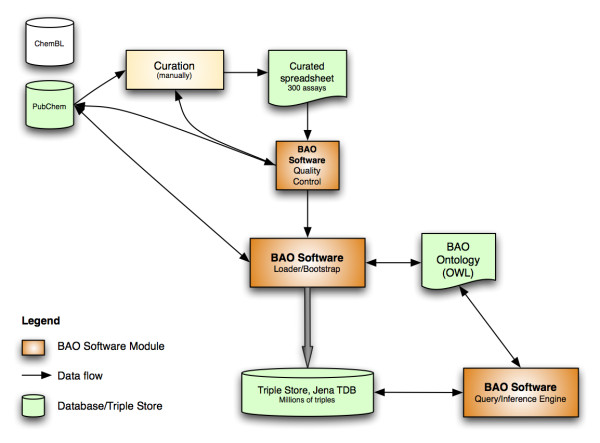
**BAO software modules (orange/dark gray), documents and databases (light green/light gray)**.

The work flow for applying the ontology to real data from PubChem is illustrated in Figure 3. First, we have summarized a set of attributes about the assays that needed to be annotated. We have considered >120 attributes (e.g., "EndpointStandardized", which takes values of IC50, percent inhibition, fold activation, etc.). These attributes are populated row-by-row in a spreadsheet for the relevant assays using a local mirror of the PubChem data source. A major portion of the spreadsheet is curated manually. In order to compensate for the errors that may have been introduced during the manual work, we have written a software module to cross-reference each entry in the spreadsheet with the PubChem data source. There were some redundant information among the annotation spreadsheet and data in PubChem, for example screening concentration reported in the assay description (which was manually curated) and the screening concentration deposited to PubChem (which was available in the mirror data source). Some information in the annotation template was explicitly repeated from PubChem in order to correctly map annotated (standardized) terms to data in PubChem, for example to standardize endpoints. This redundancy can be seen as a quality control step to uncover any discrepancies between original and curated data. This step has revealed some inconsistencies in the PubChem database, such as PubChem table entries that are not atomic, incorrect or missing screening concentrations or units; and it has also helped to minimize the errors that had been made throughout the cumbersome curation process. Second, we have developed a core software module, described as Loader/Bootstrap in Figure 3, which reads the curated and quality-checked data and then uses the ontology as well as necessary PubChem data to create a *logical *model of the domain. The reasoning engine Pellet was used, both to create and query the domain model. Pellet is a server-based OWL-DL reasoner that supports SROIQ(D). We also experimented with other DL reasoners, such as HermiT and FaCT++, but used Pellet because of its existing API (Application Programming Interface) that allows interfacing to other software components that we use.

**Figure 4:**
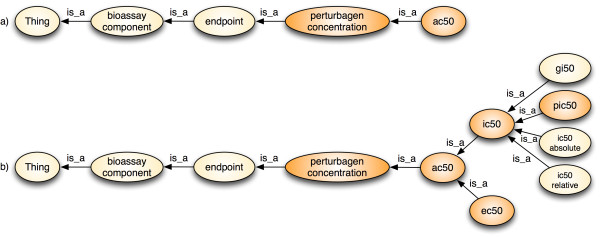
**a) Asserted logical taxonomy for AC50 (above) and b) Inferred logical taxonomy, where IC50 is classified as a sub-class of AC50**.

Of particular note here is the BAO expressivity of SROIQ(D). S allows atomic and complex concept negation, concept intersection, universal restrictions, limited existential quantification and transitive roles. R stands for limited complex role inclusion axioms; reflexivity, irreflexivity and role disjointness. O stands for nominals, I for inverse properties, Q for qualified cardinality restrictions and (D) for the use of datatype properties or data values. The reasoner checks the internal consistency of the logical model and inferred hidden knowledge. One example for this is the class AC50, which was inferred to be a superclass of IC50 (see Figure 4b). The ontology specification defines AC50 as the concentration of the perturbagen that results in either 50% activation (EC50) or inhibition (IC50). Figures 4a and 4b show the asserted and inferred models of AC50, respectively.

### Ontology-facilitated query examples

We performed a series of experiments based on 194 out of the 300 curated PubChem bioassays that had the (standardized) endpoint terms IC50, EC50, AC50, percent activation, percent stimulation and percent inhibition. Since the entire set of assays and endpoints would have required > 17 GB worth of RDF triples, we decided to limit the amount of considered endpoints to 20 for performance reasons. Future versions of the software will focus on optimization and the use of additional annotations. With 20 endpoints, the software generated 45,075 triples (asserted ontology + triple database) in the Jena store. All example queries can be found and tested online at http://baoquery.ccs.miami.edu/joseki/query.html. The reasoner classifies the individuals and SPARQL allows an efficient search through this inferred graph.

*Example 1: *This example illustrates a common query for compounds with an IC50 value of less than a certain cutoff (here ≤ 10 *μ*M). Such a query should also return results of differently named IC50 endpoints (e.g. AC50), which a user may not know exist. A user querying the database may also be interested in returning other relevant endpoints, such as IC80 values ≤10 *μ*M (if they existed in the repository) or other result types such as potent inhibitors screened at less than the IC50 concentration. With the semantic definition of IC50 above, we can achieve both. Query: return all compounds from assays with an inhibitory mode of action and that have a percentage response of 50% or greater at ≤10 *μ*M screening concentration.

The SPARQL query was the following:

PREFIX rdf: <http://www.w3.org/1999/02/22-rdf-syntax-ns#>

PREFIX rdfs: <http://www.w3.org/2000/01/rdf-schema#>

PREFIX xsd: <http://www.w3.org/2001/XMLSchema#>

PREFIX owl: <http://www.w3.org/2002/07/owl#>

PREFIX bao: <http://www.bioassayontology.org/bao#>

# results

SELECT DISTINCT ?compound ?endpoint ?type ?responseValue ?screeningConc ?assay

WHERE {

# from endpoints

?endpoint rdf:type bao:BAO _0000179.

?endpoint bao:BAO_ 0000196 ?inhibition.

# has a mode of action inhibition

?inhibition rdf:type bao:BAO _0000091.

# perturbagen concentration endpoint

?endpoint bao:BAO _0000336 ?screeningConc.

# has concentration unit micro molar

?endpoint bao:BAO_0000183 bao:BAO_0000107.

# has percent response

?endpoint bao:BAO _0000337 ?percentResponse.

?percentResponse bao:BAO_ 0000195 ?responseValue.

?endpoint rdf:type ?type.

?type rdfs:subClassOf bao:BAO_ 0000180.

# response endpoint

UNION {

?endpoint bao:BAO_ 0000196 ?inhibition.

?inhibition rdf:type bao:BAO_ 0000091.

?endpoint bao:BAO_ 0000338 ?pert.

?pert bao:BAO_0000183 bao:BAO_ 0000107.

?pert bao:BAO _0000336 ?screeningConc.

?pert bao:BAO_0000183 bao:BAO_ 0000107.

?endpoint bao:BAO_0000195 ?responseValue.

?endpoint rdf:type ?type.

?type rdfs:subClassOf bao:BAO_ 0000181.

}

?endpoint bao:BAO_0000185 ?compound.

?endpoint rdf:type ?type.

?assay bao:BAO _0000209 ?measureGroup.

?measureGroup bao:BAO_ 0000208 ?endpoint.

# screening concentration <= 10 micro molar && # percent

# response >= 50%

FILTER(?screeningConc <= 10 && ?responseValue >= 50)

}

The BAO software returns 2,741 SPARQL endpoint results from the inferred model residing in the triple store, 4 of which are shown below for illustrative purposes. All results are individuals with a working internal resource identifier (IRI), which corresponds to a URI, but is valid only internally. IRIs are abbreviated due to space limitations, but all complete IRIs are available via http://baoquery.ccs.miami.edu/joseki/query.html

(5) (?compound=<bao#individual_BAO_0000021_2858522>)

(?endpoint=<bao#individual_BAO_0000190_2_2357>)

(?type=<bao#BAO_ 0000190>)

(?responseValue="50"^^^^xsd:float)

(?screeningConc="4.0"^^^^xsd:float)

(?assay=<bao#individual_BAO_0000015_1293>)

(17) (?compound=<bao#individual_BAO_0000021_133407>)

(?endpoint=<bao#individual_BAO_0000190_2_2533>)

(?type=<bao#BAO_ 0000190>)

(?responseValue="50"^^^^xsd:float)

(?screeningConc="8.59"^^^^xsd:float)

(?assay=<bao#individual_BAO_0000015_2409>)

(24) (?compound=<bao#individual_BAO_0000021_11057>)

(?endpoint=<bao#individual_BAO_0000190_2_4122>)

(?type=<bao#BAO_ 0000186>)

(?responseValue="50"^^^^xsd:float)

(?screeningConc="6.3096"^^^^xsd:float)

(?assay=<bao#individual_BAO_0000015_948>)

(2690) (?compound=<bao#individual_BAO_0000021_657680>)

(?endpoint=<bao#individual_BAO_0000201_1_1670>)

(?type=<bao#BAO_ 0000201>)

(?responseValue="63.48"^^^^xsd:float)

(?screeningConc="4.0"^^^^xsd:float)

(?assay=<bao#individual_BAO_0000015_834>)

Results are shown by their unique IRIs, e.g. the first result contains the compound ID (CID) 2858522 [29] of an individual of the class perturbagen (BAO_0000021). The SPARQL query also selects for the endpoints of the perturbagens that fulfill the activity criteria. The query retrieves results that classify as specific types of endpoints (subsumption reasoning). Result (5) (CID 2858522, AID 1293) was found because IC50 (note, that in PubChem AID 1293 this endpoint has been incorrectly reported as EC50; we corrected this during the curation process) (BAO_0000190) *is*_*a *perturbagen concentration-type endpoint (as defined above). Result (17) (CID 133407, AID 2409) also returns IC50. Result (18) (not shown) returns the same data as AC50 concordant with the (inferred) subsumption hierarchy (compare Figure 4b). Querying AC50 (instead of IC50) thus would also retrieve this result. Result (24) (CID 11057, AID 948) is an AC50 endpoint (named "potency" in PubChem); result (23) returns the same data as IC50 (not shown) - again consistent with the inferred class hierarchy. Result (2690) (CID 657680, AID 1834) is a percentage inhibition endpoint (63.5%) and the screening concentration is 4 *μ*M (i.e. less than the query 10 *μ*M). These different types of results can be retrieved because of the subsumption reasoning of the DL engine using formally defined endpoints. This example illustrates that with the endpoint definition in BAO, we can identify and return relevant query results, which are not restricted to a specific endpoint type or endpoint representation (that is specified by the query), as it would typically be the case in a relational system.

*Example 2: *Here, we illustrate an example of constructive reasoning in identifying compounds of a particular pharmacological action. Query: return all assays with compounds that have a mode of action "activation" and show a percentage response of ≥ 50% at ≤ 10 *μ*M screening concentration. The query syntax was the following (we are omitting the PREFIX section this time):

SELECT DISTINCT ?compound ?endpoint ?type ?moaType ?responseValue ?screeningConc ?assay 

WHERE {

# from endpoints

?endpoint rdf:type bao:BAO_0000179.

?endpoint bao:BAO_ 0000196 ?activation.

# has a mode of action activation

?activation rdf:type bao:BAO_ 0000087.

?activation rdf:type ?moaType.

?moaType rdfs:subClassOf bao:BAO_ 0000084.

# perturbagen concentration endpoint

?endpoint bao:BAO_ 0000336 ?screeningConc.

# has concentration unit micro molar

?endpoint bao:BAO_0000183 bao:BAO_0000107.

# has percent response

?endpoint bao:BAO_0000337 ?percentResponse.

?percentResponse bao:BAO_0000195 ?responseValue.

?endpoint rdf:type ?type.

?type rdfs:subClassOf bao:BAO_0000180.

# response endpoint

UNION {

?endpoint bao:BAO_ 0000196 ?activation.

?activation rdf:type bao:BAO_ 0000087.

?activation rdf:type ?moaType.

?moaType rdfs:subClassOf bao:BAO_ 0000084.

?endpoint bao:BAO_0000338 ?pert.

?pert bao:BAO_0000183 bao:BAO _0000107.

?pert bao:BAO _0000336 ?screeningConc.

?pert bao:BAO_0000183 bao:BAO_ 0000107.

?endpoint bao:BAO _0000195 ?responseValue.

?endpoint rdf:type ?type.

?type rdfs:subClassOf bao:BAO_ 0000181.

}

?endpoint bao:BAO_0000185 ?compound.

?endpoint rdf:type ?type.

?assay bao:BAO _0000209 ?measureGroup.

?measureGroup bao:BAO_ 0000208 ?endpoint.

# screening concentration <= 10 micro molar && # percent

# response >= 50%

FILTER(?screeningConc <= 10 && ?responseValue >= 50)

}

Similar to example 1, the system returns different types of relevant results. In addition to assays with compounds that have an endpoint "percent activation" of 50% at <10 *μ*M, this query also returns assays with an EC50 or an AC50 value of <10 *μ*M. Moreover, this example demonstrates one of the constructive reasoning mechanisms in BAO where "activation" was defined as *equivalent *to "stimulation" (among other equivalent classes, e.g. agonist). As the reasoning system returns results that satisfy the original query and the inferred query, searching "activation" (BAO_0000087) returns exactly the same results as querying for "stimulation" (BAO_0000093) independent from the specific term used to describe the pharmacological action. Selected results are:

(1) (?compound=<bao#individual_BAO_0000021_653469>)

(?endpoint=<bao#individual_BAO_0000188_2_5524>)

(?type=<bao#BAO_ 0000180>)

(?moaType=<bao#BAO_ 0000087>)

(?responseValue="50"^^^^xsd:float)

(?screeningConc="2.154"^^^^xsd:float)

(?assay=<bao#individual_BAO_0000015_695>)

(5) (?compound=<bao#individual_BAO_0000021_653469>)

(?endpoint=<bao#individual_BAO_0000188_2_5524>)

(?type=<bao#BAO _0000188>)

(?moaType=<bao#BAO_ 0000093>)

(?responseValue="50"^^^^xsd:float)

(?screeningConc="2.154"^^^^xsd:float)

(?assay=<bao#individual_BAO_0000015_695>)

(5130) (?compound=<bao#individual_BAO_0000021_645132>)

(?endpoint=<bao#individual_BAO_0000200 1_464>)

(?type=<bao#BAO_ 0000181>)

(?moaType=<bao#BAO_ 0000087>)

(?responseValue="132.52"^^^^xsd:float)

(?screeningConc="5.7"^^^^xsd:float)

(?assay=<bao#individual_BAO_0000015_1318>)

(5131) (?Compound=<bao#individual_BAO_00000021_645132>)

(?endpoint=<bao#individual_BAO_0000200_1_464>)

(?type=<bao#BAO_ 0000200>)

(?moaType=<bao#BAO_ 0000093>)

(?responseValue="132.52"^^^^xsd:float)

(?screeningConc="5.7"^^^^xsd:float)

(?assay=<bao#individual_BAO_0000015_1318>)

The first result (1) refers to AID 695 [30]. As before, the formal definition of "mode of action" in the ontology and the reasoning system make it possible to retrieve relevant results by inference, which could not be returned from a relational database system (e.g. agonist if one searched for activation).

**Figure 5:**
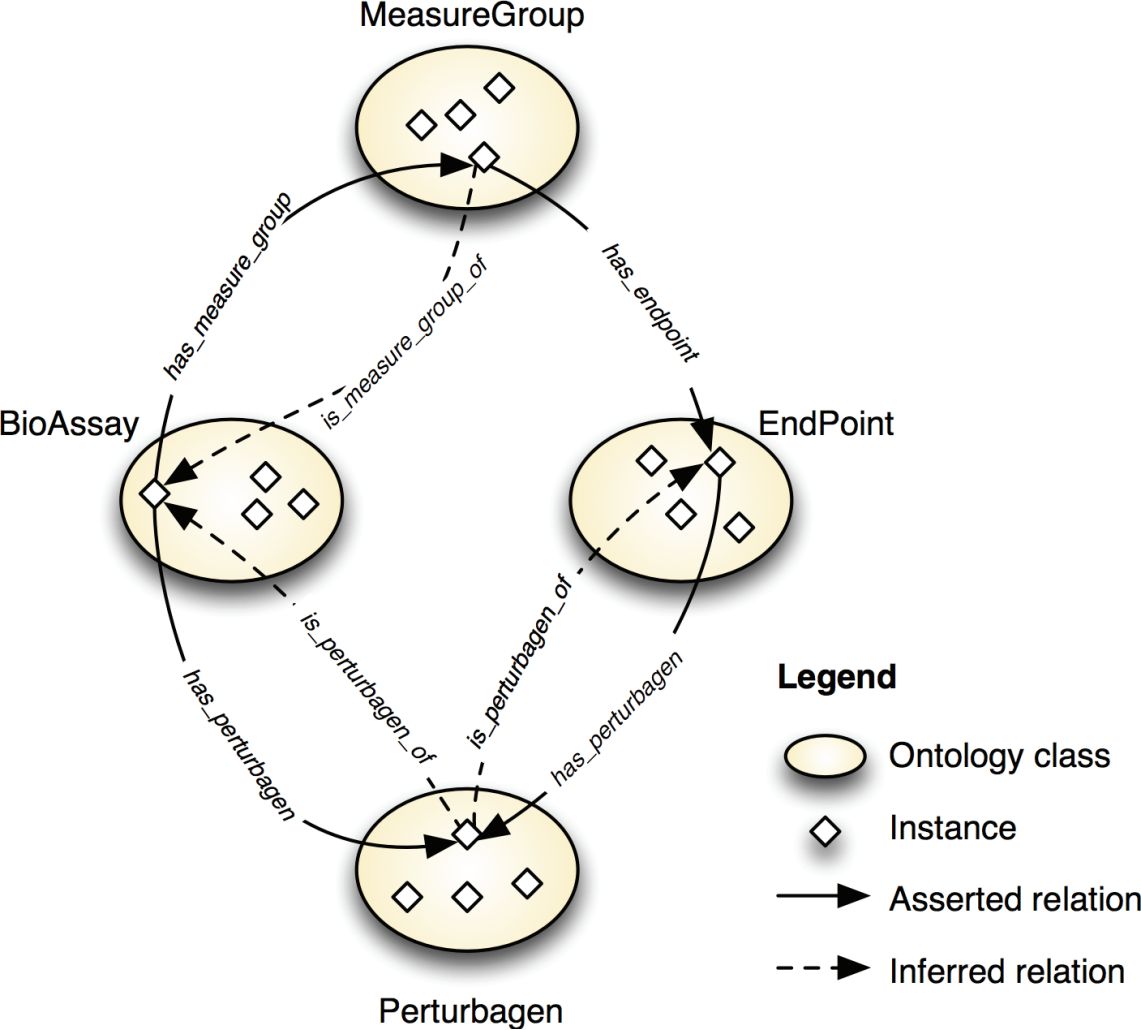
**Relationships between BioAssay, EndPoint, and Perturbagen**.

*Example 3: *With this example, we demonstrate a specific case concerning three concepts: endpoint, bioassay, and perturbagen. Figure 5 shows the relevant relationships between these concepts (note: the concept "measure group" exists to accommodate multiplexed assays; it is not used in this example); it is a more detailed representation of some of the concepts in Figure 1. Of particular interest is the relation *"has perturbagen" *that holds between endpoint and perturbagen as well as bioassay and perturbagen. The ontology specifies that this property has an *inverse *relationship with *"is perturbagen of"*. Here we show how these relationships (with their characteristics) are used to retrieve eligible instances (individuals) by inference. This reasoning mechanism thus makes it possible to retrieve perturbagens based on more complex concepts, for example a class of promiscuous compounds (compounds that are active in several assays - see below).

To illustrate this, we queried for all perturbagens that have a percentage response of ≥50% in at least three assays. The SPARQL query was as follows:

SELECT ?pert

WHERE

{ ?pert rdf:type bao:BAO_0000021.

?pert bao:BAO_0000361 ?assay.

?assay bao:BAO_0000209 ?measureGroup.

?measureGroup bao:BAO_ 0000208 ?endpoint.

?endpoint bao:BAO_0000195 ?percentResponseValue.

}    UNION

{ ?pert rdf:type bao:BAO_0000021.

?pert bao:BAO_0000361_?assay.

?assay bao:BAO _0000209 ?measureGroup.

?measureGroup bao:BAO_0000208 ?endpoint.

?endpoint bao:BAO_0000337 ?percentResponse.

?percentResponse bao:BAO_0000195 ?percentResponseValue.

}

FILTER (?percentResponseValue >= 50)

}

GROUP BY ?pert

HAVING (count(distinct ?assay) >= 3)

In this query, we used the inferred relation *"is perturbagen of"*, which points to either an endpoint or a bioassay. The query separately checked for bioassay instances and endpoint instances. The syntax allowed for the expression of the notion of "at least" in a simple way. Specifically, we used the syntactic extensions available in the ARQ SPARQL [31] implementation. The "GROUP BY" extended clause grouped the unique "?pert" result set (?pert is a variable here) in a row-by-row basis. The "HAVING" clause applied the lter "count(distinct ?assay))" to the result set after grouping. The results of the query were as follows. First, we queried for the compound and obtained:

(1) (?pert=<bao#individual_BAO_0000021_646704>)

We then used this result (bao:individual_BAO_0000021_646704) for the next query:

SELECT ?assay ?percentResponseValue

WHERE

{ bao:individual_BAO_0000021_646704 bao:BAO_0000361 ?assay.

?assay bao:BAO_0000209 ?mg.

?mg bao:BAO_0000208 ?endpoint.

bao:individual_BAO_0000021_646704 bao:BAO_0000361 ?endpoint.

?endpoint bao:BAO_0000195 ?percentResponseValue.

}    UNION

{ bao:individual_BAO_0000021_646704bao:BAO_0000361 ?assay

?assay bao:BAO_0000209 ?mg.

bao:individual_BAO_0000021_646704 bao:BAO_0000361 ?endpoint.

?endpoint bao:BAO_0000337 ?percentResponse.

?percentResponse bao:BAO_000195 ?percentResponseValue.

}

FILTER (?rv >= 50)

}

Here are the final results:

(1) (?assay=<bao#individual_BAO_0000015_1262>)

(?percentResponseValue="116.84"^^^^xsd:float)

(2) (?assay=<bao#individual_BAO_0000015_1306>)

(?percentResponseValue="106.48"^^^^xsd:float)

(3) (?assay=<bao#individual_BAO_0000015_1316>)

(?percentResponseValue="99.42"^^^^xsd:float)

Example 3 is a simple illustration to identify compounds with a specific profile (here, active in three assays). The query actually retrieved inferred information, facilitated by the *inverse *relationship *"is perturbagen of"*. Further specification of this query, e.g. by BAO meta target or design sub-classes, would allow to quickly identify individuals based on more complex concepts, for example compounds that are promiscuously active in assays of a specific design and which are therefore likely artifacts.

The three query examples illustrate some of the features that can be used in complex search queries with an underlying DL-based ontology. Other features such as role hierarchies, quantifiers, nominals etc. were also used in our ontology.

## Conclusions

We have developed an ontology to describe biological assay and screening results. The BioAssay Ontology (BAO) provides a foundation for standardizing assay descriptions and endpoints and serves as a knowledge model by describing screening experiments and results semantically using description logic (OWL language). BAO facilitates semantic search capabilities enabling the retrieval of data that are relevant to a query and that could not be readily obtained otherwise. 300 PubChem assays were curated and 194 were loaded into a triple store to demonstrate various search scenarios. The ontology was published (current version 1.2b868) and is available at http://bioassayontology.org and the NCBO bioportal. This is the first ontology to describe this domain, and certainly the first time that bioassay and HTS data have been represented using expressive description logic. There are numerous advantages to this approach; most importantly it opens new functionality for querying and analyzing HTS datasets and the potential for discovering knowledge that is not explicitly represented, by inference. We demonstrated these novel capabilities and their benefits by three simple examples of how specific features of our approach can be implemented. One of the examples illustrated a query for (inferred) perturbagens with a defined activity profile. As BAO includes class hierarchies for target, design, detection technology, etc., perturbagen sub-classes of interest may be directly defined in the ontology using the same approach; e.g. "compounds promiscuously active in luciferase reporter gene assays". Using a reasoning engine, the individuals that are members of such a class could be automatically inferred among the currently annotated assays. We are continuing to refine and extend the BAO and supporting software. We have already created a web portal with an easy-to-use querying interface that incorporates some of the described functionality [32]. A user can query PubChem data using BAO terminology and collect sets of results for further analysis. It also allows end users to formulate their own queries via a graphical user interface. Future developments will include an annotation tool for domain experts that will aid in the curation process and the incorporation of additional data sources.

## Competing interests

The authors declare that they have no competing interests.

## Authors' contributions

List of contributions: UV carried out and supervised the development of the ontology as a knowledge engineer, performed research on semantic web technologies and wrote a major part of the manuscript. SA carried out the development of the ontology as a knowledge engineer and wrote software for querying the ontology. UVe performed research on bioassays from various sources, curated the assays, developed ontology as a domain expert, and wrote parts of the manuscript. RPS developed the first versions of ontology and edited the manuscript. VL contributed to the ontology and edited the manuscript. SCS designed the research topic, performed research on the domain, developed the ontology as a domain expert, analyzed results, and wrote major parts of the manuscript. All authors read and approved the final manuscript.

## Methods

### Ontology development

The development of ontologies, the annotation of documents and data with terms from various ontologies as well as the use of ontologies can be complex, cumbersome, and confusing. Thus, researchers have spent a good portion of the last decade to develop supporting tools. They can be classified into the following three categories:

#### Ontology construction

There exist whole suites, e.g. OntoStudio, NeOn Toolkit and single editors, e.g. Protégé [33]. In addition, systems for visualizing ontologies [34] and methods to analyze ontologies [35] have been developed. Their primarily purpose is not the construction of ontologies. However, a large ontology can be very complex so that analysis/visualization tools are necessary or at least helpful throughout the development process. To construct BAO, we used Protégé version 4.1. We used OWLViz [36] for visualization and Pellet [37] as an appropriate DL reasoning engine. We used OntoFox [38] and the OWL API [39] to extract and integrate modules from external ontologies such as Gene Ontology (GO), NCBI Taxonomy, Cell Line Ontology (CLO) into BAO. Namespaces of these external ontologies were preserved.

#### Query and inference systems

There is a large variety of special query languages available [40]. Prominent query languages cover queries for the traditional web (XML data), and for the Semantic Web (i.e. mostly RDF data). Examples for the former are XPath [41] or XQuery [42]), examples for the latter are RQL [43] or SPARQL [44]). Languages targeting OWL (e.g. SWRL [45]) are becoming more and more important and will also be used in the future. However, for the work presented here our main query language was SPARQL. The reason for this choice was the vast amount of data we had to operate on. We used a RDF triple store with millions of data records. The triple store consisted of the inferred model of our domain, i.e. the asserted and inferred factual knowledge. SPARQL is currently the best query language for triple stores, because it can be used to express queries across diverse data sources, whether the data are stored natively as RDF or viewed as RDF via middleware.

Researchers can choose from a number of powerful inference engines that have been developed over the past few years. Some ontology editors such as Protégé come with integrated inference engines (Pellet), but also can be operated with alternative systems. Prominent and state-of-the-art examples are HermiT [46], Pellet, Racer [47], and FaCT++ [48]. All of them are DL reasoning engines that have been proven to be sound and complete. Differences lay in reasoning capabilities, performance and expressivity [48].

#### Further tools

These include API's, proof tools and special programs: APIs are useful if researchers would like to use existing or developed ontologies and the reasoning capabilities of the DL engines and combine them with their own software. Prominent examples are OWL API [39] for working with OWL 2 ontologies, the Thea-Prolog-OWL-Library [49] that uses SWI Prolog's RDF library or the Jena API [50], which is a Java framework for building Semantic Web applications. It provides an environment for RDF, RDFS and OWL, SPARQL and also includes a rule-based inference engine. We used the Jena-API and the Jena SDB component, because it provides means for large scale storage and queries of RDF datasets.

### Curation of assay data

In an effort to make the PubChem data amenable to large-scale computational analysis, we manually curated the bioassays. Detailed information were captured from each individual assay based on BAO classes, which fall into the main categories format, meta target, design, detection technology, perturbagen (at this point we only consider small molecule compounds), endpoint, and general assays characteristics. The annotations from each assay were populated in a spreadsheet, cross checked, and then loaded onto a triple store after merging with the relevant PubChem endpoint data using the ontology as described above. In addition to the bioassays run at the MLPCN, PubChem houses data from other sources. Most notable is the recent (October 2010) deposition of ~460,000 assay records from the ChEMBL database. We are in the process of incorporating these datasets into BAO.

### Implementation and application

**Figure 6:**
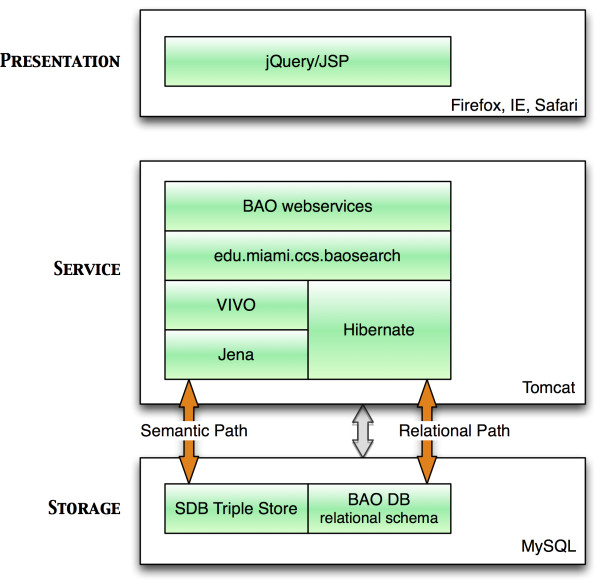
**High-level architecture of BAOSearch**.

The ontology was used to facilitate the featured search queries. Our "BAOSearch" is an application for querying, viewing, browsing and downloading diverse high-throughput screening (HTS) data for drug discovery and related life science research [32]. BAOSearch is a multi-tier, web-based, AJAX-enabled application written primarily in Java and built following a Restful [51] web services paradigm. The service-based aspect of the architecture allows the user interface (UI) to be separated from storage and manipulation of the data, and provides well-defined interfaces for UI components to access and manipulate application data. This separation of application components creates the potential of developing multiple UIs that access the same service, but which render the data differently, or run on different platforms (e.g., browsers, mobile applications). This architecture also creates an opportunity for other software applications (not only UIs) to access the system to query and retrieve data. The browser-based UI was built using JSP and JavaScript, with components from several JavaScript libraries including jQuery [52]. All data were stored in a MySQL database. SDB [53] was used as the triple-store. Other data required by the application was stored in a relational schema accessible using Hibernate. Figure 6 shows the high-level architecture of the BAOSearch project.

## Acknowledgements

This project is funded by the NIH under the grant number NHGRI (1RC2HG00566801). The authors thank Mark Southern and Christopher Mader for contributions to the project and the manuscript. We acknowledge resources from the Center for Computational Science at the University of Miami. Vance Lemmon holds the Walter G. Ross Distinguished Chair in Developmental Neuroscience.

## References

[B1] Posner BA High-throughput screening-driven lead discovery: meeting the challenges of finding new therapeutics Curr Opin Drug Discov Devel 2005 8 4 487 494 16022185

[B2] Molecular Libraries Initiative http://nihroadmap.nih.gov/molecularlibraries/[Last checked on 6/3/2011]

[B3] Austin CP Brady LS Insel TR Collins FS NIH Molecular Libraries Initiative Science 2004 306 5699 1138 1139 10.1126/science.1105511 15542455

[B4] Molecular Libraries Program http://mli.nih.gov/mli/[Last checked on 6/3/2011]

[B5] PubChem http://pubchem.ncbi.nlm.nih.gov/[Last checked on 6/3/2011]

[B6] Roy A McDonald P Sittampalam S Chaguturu R Open Access High Throughput Drug Discovery in the Public Domain: A Mount Everest in the Making Curr Pharm Biotechnol 2010 10.2174/138920110792927757 PMC3716285 20809896

[B7] EU project OpenScreen http://www.eu-openscreen.eu/[Last checked on 6/3/2011]

[B8] ChEMBL https://www.ebi.ac.uk/chembl/[Last checked on 6/3/2011]

[B9] Psychoactive Drug Screening Program (PDSP) http://pdsp.med.unc.edu/[Last checked on 6/3/2011]

[B10] Inglese J Shamu CE Guy RK Reporting data from high-throughput screening of small-molecule libraries Nat Chem Biol 2007 3 8 438 41 10.1038/nchembio0807-438 17637769

[B11] Whetzel PL Parkinson H Causton HC Fan L Fostel J Fragoso G Game L Heiskanen M Morrison N Rocca-Serra P Sansone SA Taylor C White J Stoeckert J C J The MGED Ontology: a resource for semantics-based description of microarray experiments Bioinformatics 2006 22 7 866 73 10.1093/bioinformatics/btl005 16428806

[B12] Edgar R Domrachev M Lash AE Gene Expression Omnibus: NCBI gene expression and hybridization array data repository Nucleic Acids Res 2002 30 207 10 10.1093/nar/30.1.207 11752295 PMC99122

[B13] Wang Y Bolton E Dracheva S Karapetyan K Shoemaker BA Suzek TO Wang J Xiao J Zhang J Bryant SH An overview of the PubChem BioAssay resource Nucleic Acids Res 2010 38 Database D255 66 10.1093/nar/gkp965 PMC2808922 19933261

[B14] Ashburner M Ball CA Blake JA Botstein D Butler H Cherry M Davis AP Dolinski K Dwight SS Eppig JT Harris MA Hil DP Issel-Tarver L Kasarskis A Lewis S Matese JC Richardson JE Ringwald M Rubin GM Sherlock G Gene Ontology: tool for the unification of biology. The Gene Ontology Consortium Nature Genetics 2000 25 25 29 10.1038/75556 10802651 PMC3037419

[B15] Smith B Ashburner M Rosse C Bard J Bug W Ceusters W Goldberg LJ Eilbeck K Ireland A Mungall CJ Leontis N Rocca-Serra P Ruttenberg A Sansone SA Scheuermann RH Shah N Whetzel PL Lewis S The OBO Foundry: coordinated evolution of ontologies to support biomedical data integration Nat Biotechnol 2007 25 11 1251 5 10.1038/nbt1346 17989687 PMC2814061

[B16] National Center for Biomedical Ontologies (NCBO) http://bioportal.bioontology.org/[Last checked on 6/3/2011]

[B17] Schober D Smith B Lewis SE Kusnierczyk W Lomax J Mungall C Taylor CF Rocca-Serra P Sansone SA Survey-based naming conventions for use in OBO Foundry ontology development BMC Bioinformatics 2009 10 125 10.1186/1471-2105-10-125 19397794 PMC2684543

[B18] Schürer S Vempati U Smith R Southern M Lemmon V BioAssay Ontology Annotations Facilitate Cross-Analysis of Diverse High-Throughput Screening Data Sets Journal of Biomolecular Screening 2011 16 4 415 426 10.1177/1087057111400191 21471461 PMC3167204

[B19] BioAssay Ontology Website http://bioassayontology.org [Last checked on 6/3/2011]

[B20] Guarino N Oberle D Staab S What Is an Ontology? Handbook on Ontologies 2009 1 17

[B21] OWL 2.0, World Wide Web Consortium (W3C) http://www.w3.org/TR/2009/REC-owl2-overview-20091027/[Last checked on 6/3/2011]

[B22] Sarntivijai S Ade AS Athey BD States DJ A bioinformatics analysis of the cell line nomenclature Bioinformatics 2008 24 23 2760 6 [Sarntivijai, Sirarat Ade, Alexander S Athey, Brian D States, David J R01 LM008106/LM/NLM NIH HHS/United States U54 DA021519/DA/NIDA NIH HHS/United States Research Support, N.I.H., Extramural England Bioinformatics (Oxford, England) Bioinformatics. 2008 Dec 1;24(23):2760-6. Epub 2008 Oct 10.] 10.1093/bioinformatics/btn502 18849319 PMC2639272

[B23] Unit Ontology http://bioportal.bioontology.org/visualize/45500/[Last checked on 6/3/2011]

[B24] Brinkman RR Courtot M Derom D Fostel JM He Y Lord P Malone J Parkinson H Peters B Rocca-Serra P Ruttenberg A Sansone SA Soldatova LN Stoeckert JCJ Turner JA Zheng J Modeling biomedical experimental processes with OBI J Biomed Semantics 2010 1 Suppl 1 S7 [Brinkman, Ryan R Courtot, Melanie Derom, Dirk Fostel, Jennifer M He, Yongqun Lord, Phillip Malone, James Parkinson, Helen Peters, Bjoern Rocca Serra, Philippe Ruttenberg, Alan Sansone, Susanna-Assunta Soldatova, Larisa N Stoeckert, Christian J Jr Turner, Jessica A Zheng, Jie OBI consortium England Journal of biomedical semantics J Biomed Semantics. 2010 Jun 22;1 Suppl 1:S7.] 20626927 10.1186/2041-1480-1-S1-S7 PMC2903726

[B25] OBO Relationship Ontology http://www.obofoundry.org/ro/[Last checked on 6/3/2011]

[B26] Schmidt-Schauß M Smolka G Attributive concept descriptions with complements Artificial Intelligence 1991 48 1 26 10.1016/0004-3702(91)90078-X

[B27] Spackman KA Campbell KE Cote RA SNOMED RT: a reference terminology for health care 1997 Proc AMIA Annu Fall Symp 640 644 PMC2233423 9357704

[B28] Rogers J Rector A GALEN's model of parts and wholes: experience and comparisons Proc AMIA Symp 2000 714 718 PMC2243933 11079977

[B29] NCBI Compound ID 2858522 http://www.ncbi.nlm.nih.gov/sites/entrez?db=pccompound&term=2858522 [Last checked on 6/3/2011]

[B30] NCBI Assay ID 695 http://www.ncbi.nlm.nih.gov/sites/entrez?db=pcassay&term=695 [Last checked on 6/3/2011]

[B31] ARQ SPARQL http://jena.sourceforge.net/ARQ/group-by.html [Last checked on 6/21/2011]

[B32] BAOSearch - Searching Small Molecule Bioactivity Data via the BioAssay Ontology http://baosearch.ccs.miami.edu/baosearch/[Last checked on 6/3/2011]

[B33] Noy NF Sintek M Decker S Crubezy M Fergerson RW Musen MA Creating Semantic Web Contents with Protege-2000 IEEE Intelligent Systems 2001 16 2 60 71 10.1109/5254.920601

[B34] Katifori A Halatsis C Lepouras G Vassilakis C Giannopoulou E Ontology visualization methods--a survey ACM Comput Surv 2007 39 4 10 10.1145/1287620.1287621

[B35] Robinson EH An ontological analysis of states: Organizations vs. legal persons Appl Ontol 2010 5 2 109 125

[B36] OWLViz http://www.co-ode.org/downloads/owlviz/[Last checked on 6/3/2011]

[B37] Parsia B Sirin E Pellet: An OWL DL Reasoner Tech rep 2004 University of Maryland at College Park [Presented as poster at ISWC 2004]

[B38] Xiang Z Courtot M Brinkman RR Ruttenberg A He Y OntoFox: web-based support for ontology reuse BMC Res Notes 2010 3 175 10.1186/1756-0500-3-175 20569493 PMC2911465

[B39] Bechhofer S Volz R Lord P Cooking the Semantic Web with the OWL API The SemanticWeb-ISWC 2003 2003 659 675

[B40] Bailey J Bry F Furche T Schaffert S Eisinger N, Małuszyński J Web and Semantic Web Query Languages: A Survey Reasoning Web, Volume 3564 of Lecture Notes in Computer Science 2005 Springer Berlin/Heidelberg 35 133 http://www.springerlink.com/content/32b4c7kr9tm166yl/

[B41] Clark J DeRose S XML Path Language (XPath) Version 1.0 1999 W3c recommendation, MIT, INRIA, Keio

[B42] Chamberlin D XQuery: a query language for XML SIGMOD '03: Proceedings of the 2003 ACM SIGMOD international conference on Management of data 2003 New York, NY, USA: ACM 682 682

[B43] Karvounarakis G Magganaraki A Alexaki S Christophides V Plexousakis D Scholl M Tolle K Querying the Semantic Web with RQL Computer Networks 2003 42 5 617 640 http://www.sciencedirect.com/science/article/pii/S1389128603002275 [The Semantic Web: an evolution for a revolution] 10.1016/S1389-1286(03)00227-5

[B44] Prud'hommeaux E Seaborne A SPARQL Query Language for RDF 2006 W3c recommendation, W3C

[B45] O'Connor M Knublauch H Tu S Grosof B Dean M Grosso W Musen M Gil Y, Motta E, Benjamins VR Supporting Rule System Interoperability on the Semantic Web with SWRL ISWC 2005, Volume 3729 of LNCS 2005 Galway, Ireland: Springer 974 986

[B46] Shearer R Motik B Horrocks I HermiT: a highly-efficient OWL reasoner5th International Workshop on OWL: Experiences and Directions (OWLED 2008) 2008 Karlsruhe, Germany: Universitaet Karlsruhe 10

[B47] Haarslev V Möller R Nebel B High Performance Reasoning with Very Large Knowledge Bases International Joint Conferences on Artificial Intelligence (IJCAI) 2001 1 Seattle, WA: Morgan Kaufman 161 166

[B48] Gardiner T Horrocks I Tsarkov D Automated benchmarking of description logic reasoners Proceedings of the International Workshop on Description Logics (06) CEUR 2006 189 167 174

[B49] Vassiliadis V Wielemaker J Mungall C Processing OWL2 ontologies using Thea: An application of logic programming Proceedings of the 6th International Workshop on OWL: Experiences and Directions (OWLED) 2009

[B50] McBride B Jena: Implementing the rdf model and syntax specification 2001

[B51] Fielding R Architectural styles and the design of network-based software architectures PhD thesis 2000 UC Irvine

[B52] jQuery http://jquery.com [Last checked on 6/3/2011]

[B53] Open Jena SDB http://openjena.org/SDB [Last checked on 6/3/2011]

